# Identification and Analysis of the Superoxide Dismutase (SOD) Gene Family and Potential Roles in High-Temperature Stress Response of Herbaceous Peony (*Paeonia lactiflora* Pall.)

**DOI:** 10.3390/antiox13091128

**Published:** 2024-09-18

**Authors:** Xiaoxuan Chen, Danqing Li, Junhong Guo, Qiyao Wang, Kaijing Zhang, Xiaobin Wang, Lingmei Shao, Cheng Luo, Yiping Xia, Jiaping Zhang

**Affiliations:** 1 Genomics and Genetic Engineering Laboratory of Ornamental Plants, Department of Horticulture, Institute of Landscape Architecture, College of Agriculture and Biotechnology, Zhejiang University, Hangzhou 310058, China; 22316296@zju.edu.cn (X.C.); 22216291@zju.edu.cn (J.G.); 22216279@zju.edu.cn (Q.W.); jing1998@zju.edu.cn (K.Z.); xiaobinwang@zju.edu.cn (X.W.); 12116125@zju.edu.cn (L.S.); 22216290@zju.edu.cn (C.L.); ypxia@zju.edu.cn (Y.X.); 2 Department of Landscape Architecture, School of Civil Engineering and Architecture, Zhejiang Sci-Tech University, Hangzhou 310018, China; danqingli@zju.edu.cn

**Keywords:** herbaceous peony (*P. lactiflora*), high-temperature stress, thermotolerance, reactive oxygen species (ROS), superoxide dismutase (SOD), exogenous hormones

## Abstract

The herbaceous peony (*Paeonia lactiflora* Pall.) plant is world-renowned for its ornamental, medicinal, edible, and oil values. As global warming intensifies, its growth and development are often affected by high-temperature stress, especially in low-latitude regions. Superoxide dismutase (SOD) is an important enzyme in the plant antioxidant systems and plays vital roles in stress response by maintaining the dynamic balance of reactive oxygen species (ROS) concentrations. To reveal the members of then SOD gene family and their potential roles under high-temperature stress, we performed a comprehensive identification of the SOD gene family in the low-latitude cultivar ‘Hang Baishao’ and analyzed the expression patterns of SOD family genes (*PlSODs*) in response to high-temperature stress and exogenous hormones. The present study identified ten potential *PlSOD* genes, encoding 145–261 amino acids, and their molecular weights varied from 15.319 to 29.973 kDa. Phylogenetic analysis indicated that *PlSOD* genes were categorized into three sub-families, and members within each sub-family exhibited similar conserved motifs. Gene expression analysis suggested that SOD genes were highly expressed in leaves, stems, and dormancy buds. Moreover, RNA-seq data revealed that *PlCSD1-1*, *PlCSD3*, and *PlFSD1* may be related to high-temperature stress response. Finally, based on the Quantitative Real-time PCR (qRT-PCR) results, seven SOD genes were significantly upregulated in response to high-temperature stress, and exogenous EBR and ABA treatments can enhance high-temperature tolerance in *P. lactiflora*. Overall, these discoveries lay the foundation for elucidating the function of *PlSOD* genes for the thermotolerance of herbaceous peony and facilitating the genetic breeding of herbaceous peony cultivars with strong high-temperature resistance.

## 1. Introduction

Reactive oxygen species (ROS), including superoxide radicals (O_2_·^−^),bhydroxyl radicals (·OH), hydrogen peroxide (H_2_O_2_), and singlet oxygen (^1^O_2_), are inevitable by-products of aerobic metabolism in plant cells and play a dual role in plant growth, development, and stress response [1,2]. For example, appropriate levels of ROS can act as signaling molecules to regulate the expression of downstream genes, thereby improving plants’ tolerance to stress. However, excessive accumulation of ROS can cause membrane oxidation, damage to biological macromolecules, and even cell death [3]. When plants are subjected to biotic and abiotic stresses such as extreme temperature, drought, heavy metal, and salt, the dynamic equilibrium of ROS becomes disrupted, leading to a large amount of ROS [4,5]. To adapt to adverse environmental conditions, plants have evolved complex and efficient antioxidant defense mechanisms, including antioxidant enzymes and antioxidants [6]. For instance, the frequently studied antioxidant enzymes include superoxide dismutase (SOD), catalase (CAT), ascorbate peroxidase (APX), and glutathione peroxidase (GPX). Antioxidants such as ascorbate, proline, glutathione (GSH), α-Tocopherols, and Carotenoids are also involved in the scavenging of ROS [6,7,8].

Among these antioxidants, SOD is the first line of defense as it can catalyze the toxic O_2_·^−^ to generate O_2_ and H_2_O_2_, effectively cleaning up ROS and protecting cells from harm [6]. SOD is a ubiquitous metal enzyme family, and higher plants have three SOD isoforms according to different metal cofactors: copper zinc SOD (Cu/Zn-SOD), iron SOD (Fe-SOD), and manganese SOD (Mn-SOD) [9]. Another type of SOD, nickel SOD (Ni-SOD), mainly exists in Streptomyces, cyanobacteria, and marine life, and has yet to be described in plants [10,11]. Plant SOD genes are localized in various cellular organelles and are usually related to the production site of O_2_·^−^ [12]. Previous studies have found that Cu/Zn-SODs are mainly localized in the cytoplasm, chloroplasts, and peroxisomes; Fe-SODs are localized in chloroplasts; and Mn-SODs are localized in mitochondria and peroxisomes [6,13]. Due to their essential role in the antioxidant system and plant resistance, the SOD genes family have been widely studied in many plants, such as *Arabidopsis*, tobacco, soybean, banana, tomato, upland cotton, barley, and so on [14,15,16,17,18,19,20].

Studies have reported that plant SOD genes can be induced to cope with various environmental stresses. For example, in rapeseed, eight *BnSOD* genes were found to be significantly upregulated under hormone treatments and abiotic stresses such as salt, cold, waterlogging, and drought [21]. In liquorice, SOD activity was upregulated under salt and drought stress [22]. A progressive rise in SOD activity and upregulation of *OsCu/Zn-SOD* and *OsFe-SOD* expressions were observed under drought conditions in rice [23]. Similarly, high-temperature stress-induced *AsSODs* encode functional SOD enzymes, which increase the tolerance of garlic plants [24]. Moreover, overexpression of *OsMn-SOD* and *OsCu/Zn-SOD* enhanced resistance to heat and salt stresses in rice, respectively, by increasing the detoxification capacity of ROS [25,26]. Overexpression of *Cu/Zn-SOD* in potatoes also conferred salt tolerance [27]. These results indicate a positive correlation between higher SOD activity/expression and stress tolerance. Although some plant SODs have been suggested to improve tolerance against adverse environments, particularly under salt, drought, and high-temperature stress, the thermal response mechanism of SODs in ornamentals remains unknown.

Plant hormones are signaling compounds that regulate critical aspects of growth, development, and environmental stress responses [28]. Different studies suggest that pretreating plants with phytohormones increases the expression of genes encoding ROS-scavenging enzymes such as catalase, and results in enhanced plant thermotolerance [29]. For example, the activity of antioxidant enzymes were induced by exogenous 2,4-Epibrassinolide (EBR) treatment under high-temperature stress in tomatoes [30]. The exogenous application of Abscisic Acid (ABA) increases H_2_O_2_ accumulation, which mediates ABA-induced thermotolerance by elevating ROS scavenging enzymes and antioxidant substances. In ABA biosynthesis-deficient mutant plants that lack ABA production, heat-inducible H_2_O_2_ accumulation is abolished, consequently resulting in impaired high-temperature tolerance [31]. The exogenous Methyl Jasmonate (MeJA) application has also been found to significantly improve the high-temperature tolerance of perennial ryegrass by altering osmotic adjustment, antioxidant defense, and JA-responsive gene expression [32].

Herbaceous peony (*Paeonia lactiflora* Pall.) is an excellent ornamental perennial, cultivated worldwide for its colorful flowers and high medicinal, edible, and oil values [33,34]. It could be used as potted flowers, cut flowers, and materials for gardening to create unique seasonal landscapes [35]. However, *P. lactiflora* prefers a cool climate, and high temperatures (usually exceeding 40 °C) often cause damage, particularly in low-latitude regions [36]. This issue is becoming more severe due to global warming [37]. Prolonged exposure to high temperatures can lead to yellowing and browning leaves, premature wilting of above-ground parts, and a subsequent decrease in yield due to the insufficient accumulation of photosynthates in peony plants [38,39]. These effects directly reduce its ornamental and green value during the summer and limit the popularity and application of *P. lactiflora*. Consequently, there is an urgent need to develop herbaceous peony germplasms with enhanced resistance to high-temperature stress. 

Analyzing the SOD genes in herbaceous peony could provide critical information for the genetic improvement of high-temperature stress resistance. In the present study, we performed a systematic analysis of the SOD gene family in the herbaceous peony ‘Hang Baishao’, investigating its characteristics, including physicochemical properties, protein structure, phylogenetic relationships, conserved structural domains, motif composition, and expression profiles under high-temperature stress. Additionally, we explored the expression changes of key SOD family members under high-temperature stress treated with different hormones: EBR, ABA, and MeJA, using quantitative real-time PCR (qRT-PCR) in herbaceous peony. This comprehensive analysis of the herbaceous peony SOD gene family provides a theoretical basis for improving the high-temperature tolerance of herbaceous peony cultivars.

## 2. Materials and Methods

### 2.1. Identification of PlSOD Genes in P. lactiflora

The identification of the *PlSOD* gene family was performed in two ways based on transcriptome sequencing data of the *P. lactiflora* cultivar ‘Hang Baishao’ under natural high temperatures measured by our group. First, protein sequences of the *Arabidopsis thaliana* SOD family members were downloaded from the *Arabidopsis* genome database (TAIR). These sequences were then used as seed sequences to search the *P. lactiflora* protein database for the candidate *PlSOD* using BLASTp with an e-value ≤ 1 × 10^−5^. Second, Hidden Markov Model (HMM) profiles of Cu/Zn-SOD (PF00080) and Fe/Mn-SOD (PF02777 and PF00081) were downloaded from the InterPro database. Then, the *P. lactiflora* protein database was scanned using an HMM search with TBtools-Ⅱ software [40]. For further identification, the SOD candidate proteins were scanned in the NCBI-CDD and SMART databases to remove transcripts with incomplete domains and overlapping sequences.

### 2.2. Physicochemical Characteristics and Subcellular Localization

The physicochemical characteristics of the PlSOD proteins were predicted using the ExPASY ProtParam tool [41], including the number of amino acids, molecular weight, theoretical isoelectric point (pI), instability index, and grand average of hydropathicity (GRAVY). Subcellular localization was predicted using ProtComp 9.0 (http://linux1.softberry.com/, accessed on 15 September 2024) [42] and Cell-PLoc 2.0 (http://www.csbio.sjtu.edu.cn/bioinf/Cell-PLoc-2/, accessed on 20 February 2024) [43]. Additionally, the secondary structures of *PlSODs* were analyzed through SOPMA.

### 2.3. Phylogenetic Analysis, Motif, and Conserved Domain Analysis

To investigate the phylogenetic relationships of *PlSOD* genes, SOD protein sequences from five plant species (*A. thaliana*, *Solanum lycopersicum*, *Vitis vinifera*, *Zea mays*, and *Glycine max*) were downloaded from the NCBI and Phytozome servers. Multiple sequence alignments of the total 61 protein sequences were performed using Clustal W with default parameters [44], and a phylogenetic tree was constructed using MEGA7 via the neighbor-joining (NJ) method with a bootstrap value of 1000 [45]. The iTOL online tool was used for visualization [46]. Multiple sequence alignment of *PlSODs* was performed using DNAMAN V6 software (Lynnon Biosoft, San Ramon, CA, USA) to analyze conserved structures [47]. The MEME Suite online tool was used to identify the motifs of the SOD genes family [48], and domain information was obtained from NCBI Batch-CD-search.

### 2.4. Expression Patterns of PlSOD Genes Based on RNA-Seq Data

Eighteen independent leaf samples from the ‘Hang Baishao’ at six stages under natural high temperature stress in summer were collected for transcriptome sequencing (RNA-Seq), with three replicates at each stage (Table S1). The expression levels of the *PlSOD* genes were analyzed using the RNA-seq data. The expression of all SOD genes was normalized and represented as fragments per kilo base of exon per million fragments mapped (FPKM). Based on the FPKM value, TBtools-Ⅱ was used to evaluate the time-specific expression profile of SOD genes with Log2-based expression fold-changes and to create the heatmap [40].

### 2.5. Plant Materials

The test plants were one-year-old seedlings of *P. lactiflora* ‘Hang Baishao’, a typical low-latitude herbaceous peony. Seeds of ‘Hang Baishao’ were collected from mature fruits in late summer and sown in the Perennial Flower Resources Garden of Zhejiang University in Hangzhou (E 118°21′–120°30′, N 29°11′–30°33′), Zhejiang Province, China, in July 2022. In March of the following year, the seeds germinated, and after half a year of accumulation, one-year-old roots (length, 10–12 cm; diameter, 0.8–1.2 cm) suitable for the experiment had formed and were then collected [49]. 

Four groups of the roots were then potted in a mixture of peat soil and perlite at a 1:2 ratio and moved to a glasshouse (25/15 °C, 16/8 h day/night, 2000 lx, 85% relative humidity), with regular watering and fertilizer applications on 7 October 2023. After the three leaflets of the compound leaves of the seedlings matured, healthy plants with similar height and growth conditions were selected as the biological material for testing.

### 2.6. Treatments and Sampling

Four treatments were set up (Table 1), and the leaves of the seedlings were sprayed with solutions of EBR (Solarbio, 72962-43-7, Beijing, China), ABA (Macklin, 21293-29-8, Shanghai, China), MeJA (TCI, 1101843-02-0, Tokyo, Japan), and distilled water, respectively. The exogenous hormones were dissolved in anhydrous ethanol and then diluted to the desired concentration for each treatment, ensuring that the concentration of anhydrous ethanol in the spray solution was 0.1% (*v*/*v*). Additionally, 0.1% (*v*/*v*) Tween 80 was added as a surfactant. The control group received the same volume of anhydrous ethanol and Tween 80 as the treatment groups. Each plant was sprayed at 10:00 am for three consecutive days (preferably when water droplets had just formed).

After the final spray, the seedlings were placed into a chamber (RXZ-380, Ningbo Jiangnan instruments, Ningbo, China) for the heat treatment. The temperature in the chamber was set at 42 °C (day/night); light intensity was 2000 lx; the photoperiod was 12 h/12 h; and relative humidity (RH) was 70%. Each treatment was replicated three times, with 15 seedlings per replicate. At 0, 12, 36, and 48 h after the heat treatment, three whole seedlings from the same replicate were randomly selected and combined into one sample. These samples were then rapidly frozen in liquid nitrogen and stored at −80 °C for further experiments.

### 2.7. Determination of Chl Fluorescence

The Chl fluorescence characteristics of the four groups were observed after heat treatment for 48 h using an Imaging-PAM Chl fluorescence system (Hansatech Instruments, Norfolk, UK). After 30 min dark adaptation, the basal fluorescence (*F_o_*) and the maximum quantum yield of photosystem II (*F_v_*/*F_m_*) were measured on nine leaves from three plants per treatment (three biological replicates, with three leaves per replicate) at 10.00 am. The Y(NPQ), Y(Ⅱ), and Y(NO) parameters were also measured at 48 h [33].

### 2.8. Measurements of ROS-Related Physiological and Biochemical Indices

ROS-related physiological and biochemical indices were measured using detection kits purchased from Nanjing Jiancheng Bioengineering Co., Ltd. (Nanjing, China) following the manufacturer’s instructions [33]. Specifically, the Malondialdehyde (MDA) contents were measured using the thiobarbituric acid (TBA) method according to the MDA assay kit (A003-3). The concentration of hydrogen peroxide (H_2_O_2_) was measured by spectrophotometry using detection assay kits (A064-1). For the enzyme assays, SOD activity was measured by the WST-1 method using a SOD detection kit (A001-3).

### 2.9. RNA Extraction and qRT-PCR Analysis

Total RNA was extracted using the RNAprep Pure Plant Kit (Tiangen, Beijing, China). The purity and concentration of the RNA were assessed with a NanoDrop (ND-1000) spectrophotometer (Isogen Life Science, Utrecht, The Netherlands). Reverse transcription was performed using a PrimeScript RT Reagent Kit (TaKaRa, Kyoto, Japan). qRT-PCR was conducted using TB Green^®^ Premix Ex Taq (TaKaRa) on a CFX ConnectTM Real-Time PCR Detection system (Bio-Rad, Hercules, CA, USA) and the PCR procedure was as follows: 2 min at 95 °C; 39 cycles of 5 s at 95 °C and 30 s at 55 °C; and a melting curve program of 5 s at 95 °C, 5 s at 65 °C, and 5 s at 95 °C [50]. Primers for qRT-PCR were designed according to the herbaceous peony transcriptome database from NCBI and are listed in Table S2. Each gene was normalized to the *Alpha-tubulin* (*ATUBA*) internal reference gene [51]. The relative expression levels of these genes were determined using the 2^−∆∆Ct^ method with three biological replicates [52].

### 2.10. Statistical Analysis

All experiments in this study were conducted using a completely randomized design. One-way analysis of variance (ANOVA) and Duncan’s multiple repeat comparative analysis were performed to compare differences among different indices or treatments using the SPSS statistical program (IBM Corporation, Armonk, NY, USA), with a probability value of *p* < 0.05 considered significant. GraphPad Prism 9.0 (GraphPad Software, Inc., La Jolla, CA, USA) and Tbtools Software [40] were applied for visualization of the experimental data.

## 3. Results

### 3.1. Identification of SOD Gene Family in P. lactiflora

In this study, a total of ten SOD proteins containing at least one complete SOD domain were identified in *P. lactiflora* using eight *A. thaliana* SOD (*AtSODs*) protein sequences as queries, which are displayed in Table 2. Based on domain analysis, six of these proteins containing a copper/zinc superoxide dismutase domain (Pfam: 00080) were classified into the Cu/Zn-SOD sub-family, while four proteins containing Iron/manganese superoxide dismutases alpha-hairpin (Pfam: 00081) and C-terminal (Pfam: 02777) domains were classified into the Fe/Mn-SOD sub-family (Table 2). Comprehensive statistics for the ten *PlSOD* genes were also documented in Table 2. The protein lengths ranged from 145 to 261 amino acids (aa), and molecular weight ranged from 15,318.57 to 29,972.54 Da (from *PlCSD1-3* to *PlFSD3*). The isoelectric points extended from 6.04 (*PlMSD2*) to 8.98 (*PlMSD1*). The instability index determines whether a protein is likely to be stable (≤40, probably stable; >40, probably not stable) [53].

According to the prediction of subcellular localization, the products of three Cu/Zn-SODs (*PlCSD1-1*, *PlCSD1-2*, *PlCSD2-1*) and two Fe-SODs (*PlFSD1* and *PlFSD3*) were localized to the chloroplast, while those of two Mn-SODs (*PlMSD1* and *PlMSD2*) were localized to the mitochondria. Additionally, *PlCSD1-3*, *PlCSD2-2*, and *PlCSD3* were localized to both the chloroplast and cytoplasm. Our predictions are consistent with the existing literature, which states that Cu/Zn-SODs localize in the cytoplasm and chloroplasts, Fe-SODs primarily localize in the chloroplasts, and Mn-SODs localize in the mitochondria [54].

The secondary structure prediction indicated that the PlSOD proteins are mainly composed of alpha helices, extended strands, and random coils, with beta turns present in relatively small proportions (Table 3). Specifically, the Cu/Zn-SODs are dominated by alpha helices, while Fe/Mn-SODs are predominantly composed of extended strands.

### 3.2. Phylogenetic Analysis of PlSODs

To clarify the evolutionary relationships and classification of *PlSOD* gene sub-families, a phylogenetic tree was constructed using the protein sequences of 10 *PlSODs*, eight *A. thaliana* SODs, nine *S. lycopersicum* SODs, nine *V. vinifera* SODs, 12 *Z. mays* SODs, and 13 *G. max* SODs (Table S3). Moreover, given that orthologs often retain equivalent functions throughout evolution [55], the orthologous relationships between SODs from *A. thaliana* and *P. lactiflora* were examined using the Clustal Omega Multiple Sequence Alignment Tool at EMBL-EBI (Table S4).

**Figure 1:**
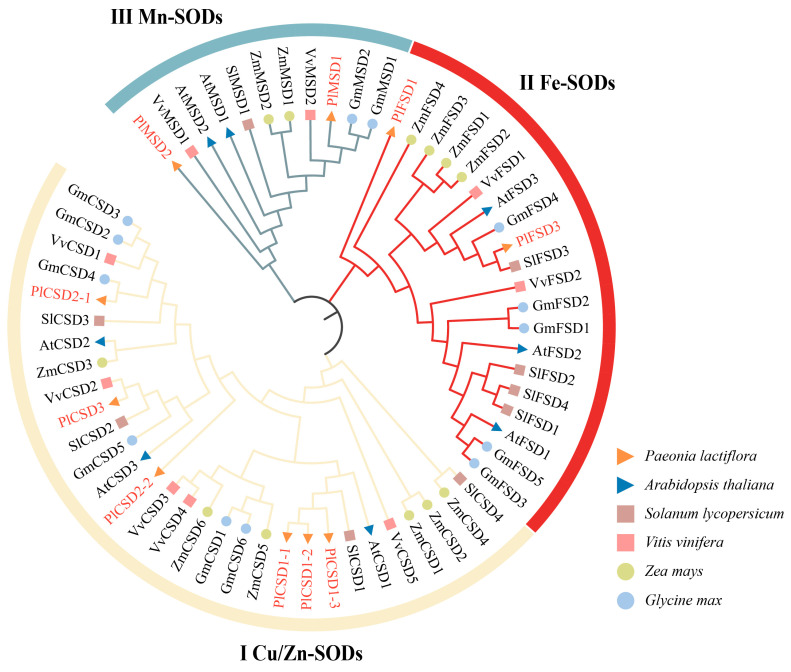
Phylogenetic tree of SOD proteins in *P. lactiflora*, *A. thaliana*, *S. lycopersicum*, *V. vinifera*, *Z. mays*, and *G. max* constructed using the neighbor-joining method. The tree was clustered into three major groups (Cu/Zn-SODs, Fe-SODs, and Mn-SODs), denoted by different colors. The proteins of herbaceous peony are marked in red and the SODs from different species were distinguished with different colors and shapes.

Based on domain analysis and the phylogenetic tree, the SOD genes of all species were clustered into three major clades: Cu/Zn-SODs (Ⅰ), Fe-SODs (Ⅱ), and Mn-SODs (Ⅲ), represented by blue, red, and light yellow, respectively (Figure 1). The Cu/Zn-SOD group contained 30 SOD members (6 *PlSODs*, 3 *AtSODs*, 4 *SlSODs*, 5 *VvSODs*, 6 *ZmSODs*, and 6 *GmSODs*), the Fe-SOD included 20 members (2 *PlSODs*, 3 *AtSODs*, 4 *SlSODs*, 2 *VvSODs*, 4 *ZmSODs*, and 5 *GmSODs*), and the Mn-SOD group had 11 members (2 *PlSODs*, 2 *AtSODs*, 1 *SlSODs*, 2 *VvSODs*, 2 *ZmSODs*, and 2 *GmSODs*). Interestingly, Cu/Zn-SOD and Fe-SOD groups contained more SODs compared to the Mn-SOD group. Additionally, sequence similarities among the six Cu/Zn-SOD proteins ranged from 51.63% to 88.24% when compared to the *AtCSD1*, *AtCSD2*, and *AtCSD3* orthologs. Notably, *PlCSD1-1* and *PlCSD1-2* shared 82.24% homology with *AtCSD1*. In the Fe-SOD group, *PlFSD1* had 56.50% similarity with *AtFSD1*, and *PlFSD3* had 72.09% similarity with *AtFSD3*. The Mn-SOD group included *PlMSD1* and *PlMSD2*, which showed 75.98% and 36.16% homology with *AtMSD1* and *AtMSD2*, respectively. Additionally, *PlCSD1-1* and *PlCSD1-2* displayed 99.48% sequence similarity, indicating they may be paralogs resulting from gene duplication. This gene duplication could be a crucial factor for diversification and functional divergence in duplicated genes, and may contribute to molecular innovation in higher organisms [56].

### 3.3. Phylogeny and Conserved Motifs of PlSOD Genes

**Figure 2:**
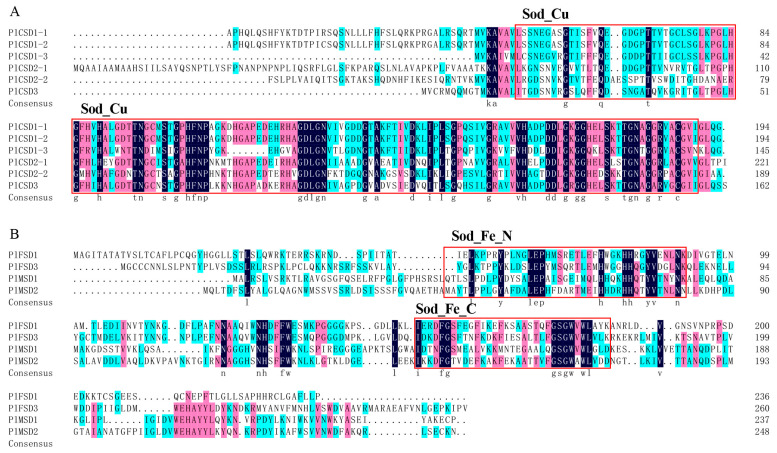
Multiple alignment of PlSOD proteins of functional domains. (**A**) Cu/Zn-SODs sub-family sequence alignment. (**B**) Fe-SODs and Mn-SODs sub-family sequence alignment. The SOD domains Sod_Cu, Sod_Fe_N, and Sod_Fe_C are marked in red boxes. The black, red, and blue parts represent homology equal to 100%, greater than 75%, and greater than 50%, respectively.

To further investigate the conserved regions among the ten members in *P. lactiflora*, multiple protein sequence alignments and pairwise identity analyses were performed. The highly conserved Sod_Cu domain ranged from approximately 132 to 141 aa, the Sod_Fe_N domain ranged from 82 to 89 aa, and the Sod_Fe_C domain spanned from 64 to 107 aa. Additionally, the ten PlSOD proteins exhibited low homology with each other, while the similarity within each sub-family was higher (Figure 2). In particular, Cu/Zn-SODs shared 44.14 to 99.48% identity, and Fe/Mn-SODs shared 23.41 to 41.18% identity (Table S5).

**Figure 3:**
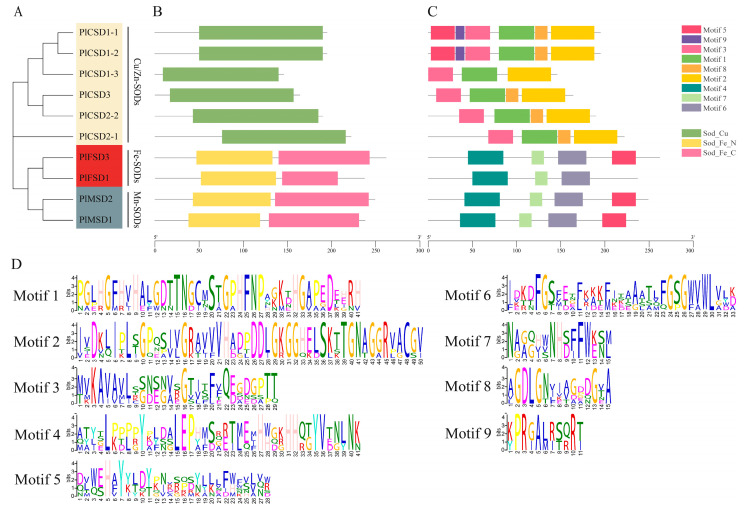
Phylogenetic tree, conserved motifs, and domain analysis of *PlSOD* genes. (**A**) Phylogenetic tree of *PlSOD* genes. (**B**) Conserved structure domains of PlSOD proteins. (**C**) Nine conserved motifs of PlSOD proteins marked by different colors. The green, yellow, and pink rectangles indicate the SOD_Cu structural domain (PF00080), SOD_Fe_N structural domain (PF00081), and SOD_Fe_C structural domain (PF02777), respectively. (**D**) Amino acids sequence of the motifs. The letter size indicates the degree of conservation in the sequences.

The ten *PlSODs* were divided into two groups (Figure 3A): one group consisted of six Cu/Zn-SODs, while the other group included four proteins: two Fe-SODs and two Mn-SODs. Analysis of conserved domains indicated that *PlFSDs* and *PlMSDs* have similar domain compositions, both containing the Sod_Fe_C and Sod_Fe_N domains. In contrast, *PlCSDs* have only a single Sod_Cu domain (Figure 3B). Conserved motif analysis further revealed the structural features of *PlSODs*. Nine conserved motifs were identified (Figure 3C), and their amino acid sequences are detailed in Figure 3D. Motifs 1, 2, 4, and 6 constitute the key functional domains of SODs mentioned above. Among these, motifs 1, 2, and 3 were presented in all six Cu/Zn-SODs, while motifs 4, 6, and 7 were shared in the four Fe/Mn-SODs. Except for *PlCSD1-3*, motif 8 was a common conserved motif in the Cu/Zn-SODs sub-family. Furthermore, motif 9 only existed in *PlCSD1-1* and *PlCSD1-2*, and motif 5 was the only motif common to both Cu/Zn-SODs (*PlCSD1-1*, *PlCSD1-2*) and Fe/Mn-SODs (*PlFSD3*, *PlMSD1*, *PlMSD2*). These results indicate that members within the same sub-family exhibit high similarity in motif compositions. However, no common motifs were shared by all ten *PlSOD* genes, further confirming the functional diversity of SOD genes in herbaceous peony.

### 3.4. Expression Analysis of PlSOD Genes in Different Tissues

**Figure 4:**
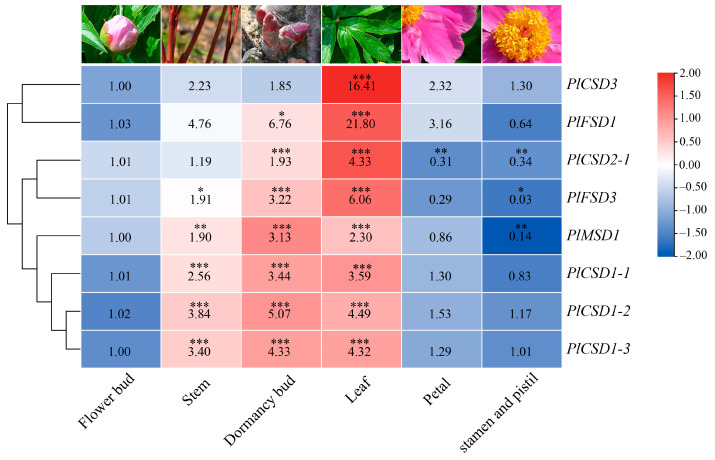
Expression analysis of *PlSOD* genes in different tissues. The expression data were obtained from qRT-PCR, and the comparisons were based on the expression levels at the flower bud. Statistically significant differences are indicated using asterisks (Duncan’s test, * *p* < 0.05, ** *p* < 0.01, and *** *p* < 0.001). Data are presented as the means ± SD of three replicates.

qRT-PCR was used to analyze the relative tissue-specific expression levels of ten *PlSOD* family members in flower bud, stem, dormancy bud, leaf, petal, stamen, and pistil of herbaceous peony under normal growth conditions. The expression of ten *PlSOD* genes was detected, with nearly all of them being highly expressed in stems, dormancy buds, and leaves, particularly in leaves, and showing low expression in flower buds, petals, stamens, and pistils (Figure 4). However, the *PlCSD2-2* and *PlMSD2* could not be detected in any of the six tissues, indicating that their expression levels were very low. Notably, *PlCSD3* and *PlFSD1* exhibited strong expression levels in leaves, with extremely significant differences. Additionally, no significant differences were observed for *PlCSD3* in other tissues. Based on these results, we chose leaves as the experimental material for follow-up observations and sampling.

### 3.5. Expression Analysis of PlSODs under Natural High-Temperature Stress

**Figure 5:**
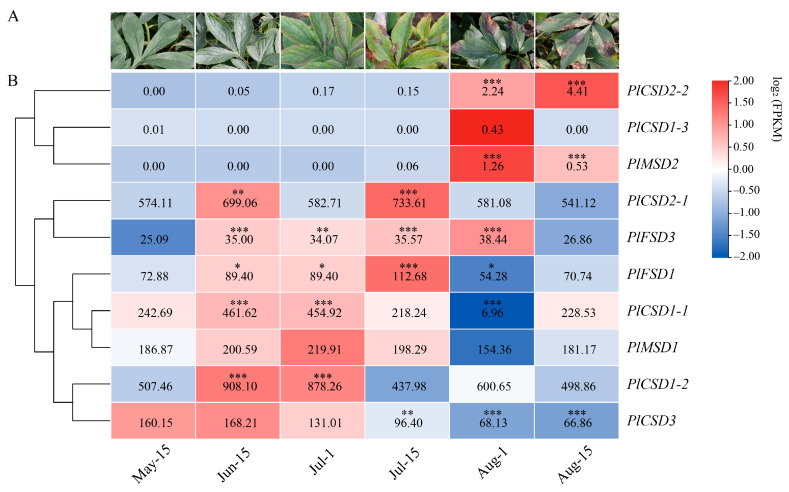
Expression profiles of *PlSOD* genes under natural high-temperature stress. (**A**) The phenotypes of herbaceous peony leaves subjected to high-temperature stress. (**B**) Expression profiles of *PlSOD* genes under high-temperature stress. The FPKM values of genes in samples were shown by different colored rectangles and the comparisons were based on the FPKM values on May 15th. Red indicates high expression. Statistically significant differences are indicated using asterisks (Duncan’s test, * *p* < 0.05, ** *p* < 0.01, and *** *p* < 0.001). Data are presented as the means ± SD of three replicates.

To investigate the potential functions of SOD genes in herbaceous peony under summer high-temperature stress, we analyzed their expression at six stages from May 15 to August 15 (May 15, June 15, July 1, July 15, August 1, August 15) in leaves using RNA-seq data. We then conducted a significance analysis of differences compared with stage one. The expression levels of herbaceous peony SOD genes at different stages exhibited substantial variations, and most *PlSOD* genes showed significant (*p*-value ≤ 0.05) changes under high-temperature stress (Figure 5). For example, *PlCSD1-1*, *PlCSD1-2*, *PlCSD3*, and *PlFSD1* were upregulated initially and then decreased. *PlCSD1-3*, *PlCSD2-2*, and *PlMSD2* exhibited extremely low expression levels compared to others, with no expression detected in the first four stages, though they increased in the fifth stage and then decreased. Additionally, the expression levels of *PlCSD2-1*, *PlFSD3*, and *PlMSD1* varied from high to low across all stages under high-temperature stress, showing no clear pattern. The above results indicated that *PlCSD1-1*, *PlFSD1*, and *PlCSD3* may play roles in the response to high-temperature stress. Overall, the herbaceous peony SOD genes may display potential divergent functions throughout plant growth and development.

### 3.6. Phenotypic and Physiological Responses to High-Temperature Stress

**Figure 6:**
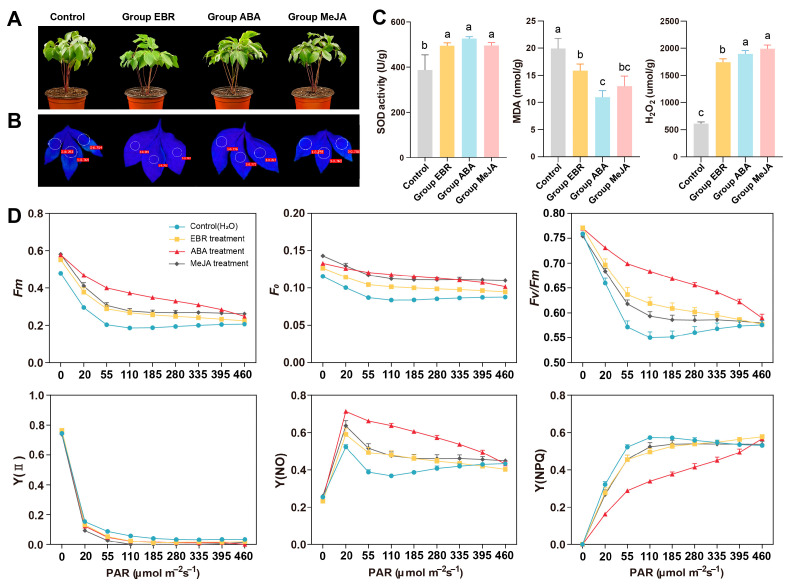
Phenotype, physiological indices, and Chl fluorescence of *P. lactiflora* plants treated with different hormones after high-temperature treatment for 48 h. (**A**) Phenotype of control group and hormone-treated group. (**B**) Chl fluorescence imaging screens. (**C**) SOD activity, MDA, and H_2_O_2_ accumulation. (**D**) Chlorophyll fluorescence parameters. All data are the means of three replicates with standard deviations, and different letters indicate significant differences among the data according to Duncan’s multiple range test (*p* < 0.05).

To explore the effects of different hormone treatments on the high-temperature resistance of *P. lactiflora*, two-week-old peony seedlings were divided into four treatment groups: Control, Group EBR, Group ABA, and Group MeJA (Table 1). There were significant phenotypic differences between the hormone-treated groups and the control group. The leaves of control plants exhibited significant shrinking, curling, and drooping, whereas the hormone-treated groups grew normally, with group EBR and group ABA performing better than group MeJA (Figure 6A). The *F_v_*/*F_m_* of the control group was also lower than that of the hormone-treated groups (Figure 6B). As shown in Figure 6C, the SOD activity in the hormone-treated groups was significantly higher than in the control group, indicating that exogenous EBR, ABA, and MeJA treatments could increase SOD activity and reduce ROS accumulation, thereby enhancing the plants’ resistance to high-temperature stress. Additionally, the MDA content was significantly lower in the treated groups than in the control. However, the H_2_O_2_ content in treatment groups was significantly higher than in the control group after high-temperature stress.

Chlorophyll fluorescence parameters reflect the plants’ adaptability to external environments and are important criteria for measuring plants’ stress-resistance [57]. The fluorescence origin (*F_o_*), fluorescence maximum (*F_m_*), the maximum quantum yield of photosystem II (*F_v_*/*F_m_*), and quantum efficiency of photosystem Ⅱ (YⅡ) values of all groups initially decreased and then stabilized, while the quantum yield of regulated energy dissipation (Y(NPQ)) and quantum yield of non-regulated energy dissipation (Y(NO)) showed the opposite trend (Figure 6D). After high-temperature stress, the *F_o_* and *F_m_* values of hormone-treated plants were higher than those of control plants, with the ABA-treated plants showing the highest parameters. Additionally, the *F_v_*/*F_m_* value, reflecting the potential activity of plant PS Ⅱ, was higher in treated plants, indicating less stress compared to controls. In this study, the *F_v_*/*F_m_* of treated plants after high-temperature stress was higher than that of control plants, indicating that the treated plants experienced less stress. The Y(NO) of treated plants was higher than that of the control, while the Y (NPQ) was lower compared to the control.

### 3.7. Exogenous Hormones and High-Temperature Stress-Induced Expression Profiles of PlSOD Family Genes

**Figure 7:**
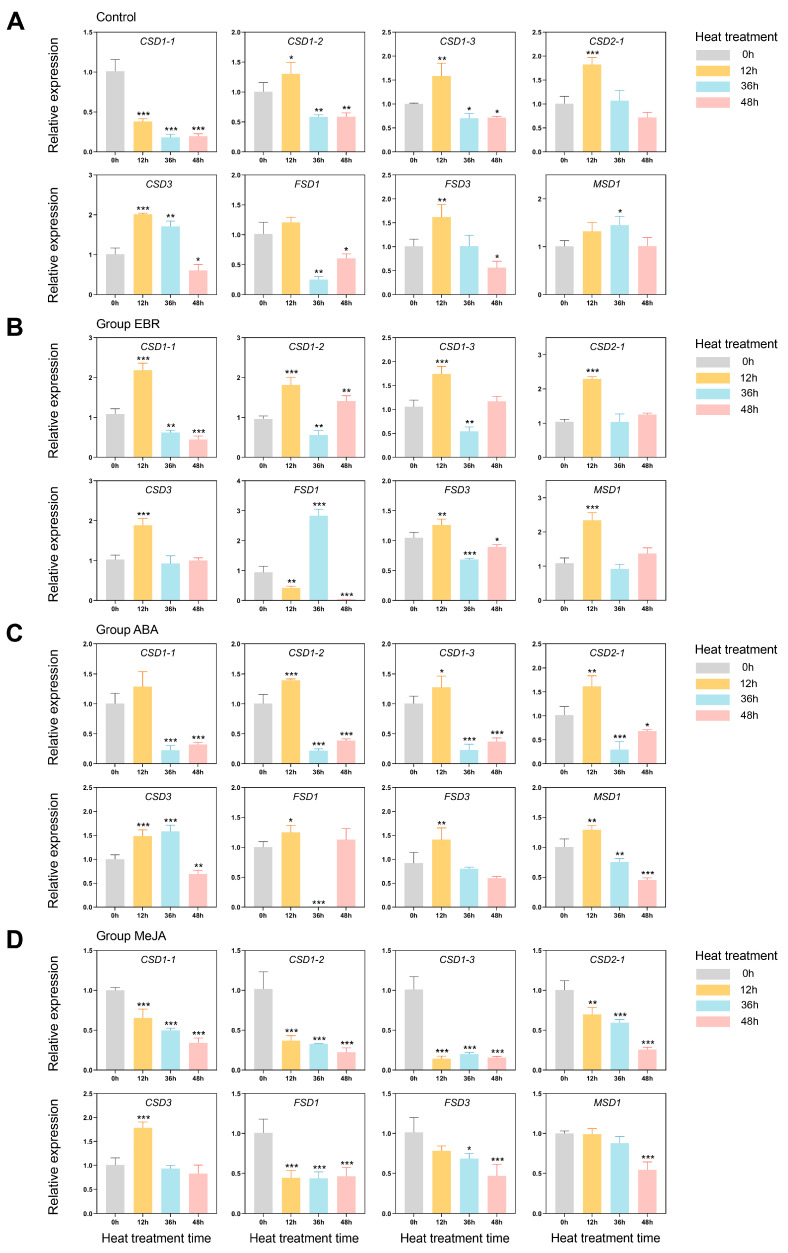
Expression profiles of *PlSOD* genes in peony leaves treated with distilled water (**A**), exogenous EBR (**B**), ABA (**C**), and MeJA (**D**) under high-temperature stress. EBR: 2, 4-epibrassinolide; ABA: Abscisic acid; MeJA: Methyl jasmonate. The mean values were derived from three independent biological replicates. ANOVA was used to test significance. * *p* < 0.05, ** *p* < 0.01, and *** *p* < 0.001. Error bars represent the standard deviation.

To further investigate the potential roles of the *PlSOD* gene family in response to high-temperature stress and the effects of different hormones on *PlSOD* expression in herbaceous peony, we selected and examined the relative expression levels of eight *PlSOD* genes after various hormone treatments and subsequent high-temperature stress at 42 °C for 0 h, 12 h, 36 h, and 48 h using qRT-PCR (Figure 7). Most *PlSOD* genes were induced by exogenous hormones, and significant transcriptional changes were observed under high-temperature stress. In the Control, EBR, and ABA treatment groups, *PlSOD* genes generally showed a trend of increasing expression followed by a decrease. Conversely, in the MeJA treatment group, most *PlSOD* genes exhibited decreased expression during high-temperature treatment. Notably, the EBR treatment group displayed a stronger response compared to the others (Figure 7).

In the Control group, the expression of most SOD genes, except for *PlCSD1-1* and *PlFSD1*, was significantly upregulated during high-temperature stress before decreasing after reaching peak levels. Specifically, the expression of *PlCSD1-1* was significantly downregulated, while *PlFSD1* initially increased slightly in the second stage before showing significant downregulation in the third and fourth stages (Figure 7A). These results indicated that, while most SOD family members could respond to high temperatures, the plant’s defense system struggles to maintain its normal state over time, leading to a decrease in SOD gene expression.

In Group EBR, all *PlSOD* genes were highly induced. With the exception of *PlFSD1*, which first decreased and then peaked with an expression level 3.02 times higher than at 0 h, the expression levels of other *PlSOD* genes increased initially before declining, peaking in the second stage (Figure 7B). Additionally, the upregulation of most SOD genes in the EBR group was notably higher than in the control group.

*PlSOD* genes in Group ABA exhibited similar expression patterns, which increased first and then decreased. *PlCSD3* reached its peak expression in the third stage, while other genes peaked in the second stage, indicating that ABA treatment might prolong the response of *PlCSD3* to high-temperature stress. All genes, except for *PlCSD1-1*, were significantly upregulated. (Figure 7C).

It is notable that, in seedlings treated with MeJA under high-temperature stress, all SOD genes except *PlCSD3* were markedly downregulated. *PlCSD3*, however, was significantly upregulated at the second stage and subsequently decreased (Figure 7D), indicating it may be influenced by MeJA signal transduction. In conclusion, the qRT-PCR data confirm that herbaceous peony SOD genes are likely involved in high-temperature stress responses. Moreover, EBR and ABA treatments may enhance the plant’s high-temperature stress response by inducing SOD genes expression.

## 4. Discussion

Herbaceous peony is a prominent landscape plant widely cultivated around the world. However, with the intensification of global warming, *P. lactiflora* is increasingly subjected to summer high-temperature stress, particularly in low-latitude regions. Such high-temperature stress interrupts physiological thermostability and triggers biochemical responses critical for plant survival [58], significantly impacting its cultivation and application [39]. SODs are crucial enzymes involved in oxidation processes, effectively reducing oxidative damage by scavenging reactive oxygen species produced under stress conditions [7]. The essential roles of SOD genes in plant acclimation to various abiotic stresses, including cold, drought, heat, and salinity, have been demonstrated in many previous studies [17,21,59]. However, detailed information on the characteristics and functions of *PlSODs*, particularly their roles in stress responses of *P. lactiflora*, remained limited. Therefore, this study conducted a systematic analysis of the *PlSOD* gene family and examined the impact of exogenous hormone treatments on the high-temperature resistance of herbaceous peony.

### 4.1. Characteristics and Evolutionary Analysis of PlSODs in P. lactiflora

In this study, a total of ten PlSOD genes were identified based on transcriptome data (Table 1). The number of SOD members in *P. lactiflora* was similar to that in *A. thaliana* (8), *S. lycopersicum* (9) [17], and *V. vinifera* (10) [60], but less than that in *Gossypium hirustum* (18) [16] and *Triticum aestivum* (26) [61]. The variety in SOD genes among different species could be due to differences in genome sizes. The results of subcellular localization of SOD proteins revealed that Cu/Zn-SODs and Fe-SODs are likely expressed in the cytoplasm and chloroplasts, while Mn-SODs are expressed in mitochondria. This distribution allows them to collaborate in maintaining the balance of free radicals in cells by functioning in different cellular compartments.

The phylogenetic relationship is based on domain analysis, with proteins clustered into the same clade generally possessing similar conserved motifs and biological functions. In this study, we constructed a phylogenetic tree of SOD proteins in *P. lactiflora* and five other species (Figure 1). These proteins were categorized into three sub-families, consistent with previous research [62,63]. Fe-SODs and Mn-SODs from different plants clustered together and were separated by a high bootstrap value, indicating that they might have originated from common ancestral genes [64]. However, as the latest enzyme in the evolutionary history of SOD, the Cu/Zn-SODs mostly existed in eukaryotes and evolved independently. At the same time, the existence of certain motifs, such as 1, 2, and 3 in all Cu/Zn-SODs and 4, 6, and 7 in all Fe/Mn-SODs, suggested that different sub-families have conserved their structures during evolution. Furthermore, the similarity between Cu/Zn-SODs and Fe/Mn-SODs showed considerable divergence. Overall, the obvious differences in motif structure and conserved domains of Cu/Zn-SODs and Fe/Mn-SODs suggest that SOD genes have a high level of function diversity during the growth and development of herbaceous peony.

### 4.2. PlSODs Are Widely Involved in High-Temperature Stress Response

Differences in expression levels and temporal and spatial specificity of a gene family are often associated with functional differentiation [12]. Based on the qRT-PCR data, we found that, except for the low expression of *PlCSD2-2* and *PlMSD2* in all tissues, the expression levels of other SOD family genes were higher in stems, leaves, and dormant buds, but lower in flower buds, petals, pistils, and stamens (Figure 4). These results suggest that SOD family genes may be involved in the growth and development of stems, leaves, and buds, especially in regulating the phenotypic changes of stems and leaves under environmental stresses, such as stalk collapse and leaf scorching. Notably, the expression levels of *PlCSD3* and *PlFSD1* in leaves were 16.41 and 21.8 times higher than those in flower buds, respectively. Leaves play a fundamental role in maintaining the life of plants through photosynthesis [65], and therefore these SOD genes may participate in scavenging the ROS generated from photosynthesis. Additionally, according to the FPKM values of 10 *PlSOD* genes at different periods, we found that *PlCSD1-1*, *PlCSD1-2*, *PlCSD3*, and PlFSD1 all showed a trend of first increasing and then decreasing, indicating that they could respond to high-temperature stress (Figure 5). However, due to the limited regulatory effect, the expression levels of all genes decreased as the stress gradually increased. In summary, the *PlSOD* genes exhibited spatiotemporally specific expression patterns.

Previous studies have reported that the main function of SOD genes is to respond extensively to various abiotic stresses [14,66,67]. For example, in bottle gourd (*Lagenaria siceraria*) almost all *LsiSOD* genes were upregulated during heat treatment [68]. Similarly, all SOD genes exhibited significant upregulation in tomato when subjected to salt and drought treatments [17]. Among these stresses, temperature is one of the most important environmental factors that affect plant growth and development. Subtle changes in temperature might impact plants morphologically, physiologically, biochemically, and molecularly, especially high temperatures in summer [38]. Indeed, high-temperature stress is now a critical issue worldwide, as it inhibits plant growth and development, and severely reduces crop yield [69,70]. For ornamental plants, their growth performance, ornamental value, and productivity are also seriously affected by high temperatures [71,72]. Studies of wheat (*Triticum aestivum*) seedlings showed that the thermotolerance to high-temperature stress was enhanced by improving the expression of chloroplast Cu/Zn-SOD and mitochondrial Mn-SOD genes [73]. Moreover, high-temperature stress strongly induced the activities of Cu/Zn-SODs, Fe-SODs, and Mn-SODs in garlic leaves [24]. In this study, eight *PlSOD* genes were significantly upregulated by high-temperature treatment, with *PlCSD2-1* and *PlCSD3* showing the most significant increases (Figure 7A), indicating that these genes may play vital roles in the high-temperature stress response. In the future, we will further verify whether these genes can regulate the thermotolerance of herbaceous peony and explore their specific regulatory mechanisms.

### 4.3. EBR and ABA Treatments Can Enhance the Tolerance of P. lactiflora under High-Temperature Stress

Recently, studies have found that the exogenous application of hormones significantly ameliorates heat-induced damage and improves plants’ high-temperature tolerance [74]. Among them, EBR, ABA, and MeJA are well-known plant growth regulators that mediate adaptations to environmental conditions [75,76,77]. In this study, we treated the herbaceous peony seedlings with EBR, ABA, and MeJA, respectively, and then measured SOD enzyme activity, and the MDA and H_2_O_2_ content, of different groups under 42 °C high-temperature stress (Figure 6). Additionally, the expression patterns of *PlSOD* genes were tested (Figure 7).

Biochemical and physiological consequences following high-temperature stress include excess accumulation of reactive oxygen species (ROS) and increased membrane permeability [78,79]. ROS can react with unsaturated fatty acids on cell membranes, leading to substantial MDA production, which exacerbates cell biofilm oxidation and disrupts its structure [80]. Therefore, the MDA levels in plant cells can indicate the degree of oxidative stress. This study showed that the activity of SOD enzyme in the hormone-treated group was higher than that of the control, while the content of MDA was lower than that of the control (Figure 6C). This indicates that exogenous hormone treatment could enhance the ROS scavenging ability of plant cells, maintain low membrane lipid peroxidation, and further reduce plant damage under high-temperature stress. Numerous studies have also been conducted to test the changes in the levels of different SOD isozymes under various abiotic stresses. For example, salt stress increased the activity of Cu/Zn-SOD in the leaves of *Citrus limonum*, and a low temperature increased SOD and catalase activity in an *Avena nuda* plant. Additionally, H_2_O_2_ content in the four groups significantly increased under heat treatment because SODs remove O_2_·^−^ by catalyzing its dismutation and produce H_2_O_2_, which possibly acts as a signal to rapidly promote the expression of stress-response proteins [81]. The photosynthetic system is highly sensitive to high-temperature stress, which induces various forms of damage, ranging from attenuating the photosynthetic rate to eliminating photosynthetic capacity [82,83]. Chlorophyll fluorescence is now widely used to monitor the photosynthetic performance of plants, especially the parameter *F_v_*/*F_m_*, which has been widely used to reflect the tolerance of plants to environmental stresses [37,84,85]. The *F_v_*/*F_m_* value of plants in the hormones-treated group was significantly higher than that in the control group after 48 h (Figure 6D), indicating that spraying EBR, ABA, and MeJA could alleviate the damage to PS II of *P. lactiflora* leaves at high temperatures, improve photosynthetic efficiency, and thus enhance the plants’ high-temperature resistance.

Our qRT-PCR results showed significant differences in *PlSOD* gene expression across different treatment groups. After EBR treatment, all the genes were upregulated from 0 to 12 h under high-temperature stress, with the upregulation trend of five genes (*PlCSD1-1*, *PlCSD1-2*, *PlCSD1-2*, *PlCSD2-1*, and *PlMSD1*) being significantly higher than that of the control, especially PlFSD1. It reached the maximum expression level at 36 h under high temperature, indicating that EBR prolonged its response time. The expression pattern of *PlSOD* family genes in the ABA-treated group was similar to that in the EBR-treated group under high-temperature stress. The difference was that the expression level of *PlCSD3* reached the highest at 36 h, while the expression level of *PlFSD1* decreased to the lowest at 36 h. It is worth noting that, in the MeJA group, almost all *PlSOD* genes were inhibited during high-temperature stress, and their expression levels gradually decreased with continued high temperature exposure. Only the expression level of *PlCSD3* was significantly upregulated after 12 h of high temperature treatment, and then it decreased. This suggests that *PlCSD3* may be involved in the ABA and MeJA pathways to regulate plants’ high-temperature tolerance. These discoveries offer robust evidence that *PlSOD* genes have different mechanisms of action and are regulated by diverse upstream factors. Moreover, these genes are involved in the high-temperature stress response, possibly via a hormone-dependent signaling pathway.

## 5. Conclusions

The current study identified ten *PlSOD* genes in herbaceous peony through RNA-seq analysis under natural high-temperature conditions. To boost our understanding, phylogenetic relationships, conserved motifs and domains, tissue-specific expression, and differential expression of these genes under natural high temperatures have been performed. Furthermore, we confirmed the expression profiles under high-temperature stress by different hormone treatments using qRT-PCR and measured the physiological indices of peony leaves post-treatment. The results revealed that several genes significantly respond to both hormonal and high-temperature stimuli, thereby enhancing our understanding of *PlSOD* genes. Thus, these genes can be targeted for breeding improvement. Our findings provided valuable insights into the SOD gene family in herbaceous peony and laid the framework for further exploring the molecular mechanisms underlying SOD gene responses to high-temperature stress. Moving forward, further validation of these genes’ functions using homologous or heterologous approaches will be a key part of subsequent work, which will help to solidify our understanding and potentially pave the way for practical applications in plant breeding and stress-tolerance enhancement.

## Figures and Tables

**Table 1:** Experimental design.

	Distilled Water Treatment	1 µM EBR Treatment	100 µM ABA Treatment	100 µM MeJA Treatment
High-temperature treatment (42 °C)	Control	Group EBR	Group ABA	Group MeJA

**Table 2:** The characteristics of SOD family proteins in *Paeonia lactiflora*.

Gene Name	Gene ID	Amino Acid	Molecular Weight	PI	Instability Index	GRAVY	Subcellular Location	Pfam Domain
*PlCSD1-1*	Peony_Unigene_344154	194	20,123.57	7.38	28.85	−0.28	Chl	CZ
*PlCSD1-2*	Peony_Unigene_468541	194	20,070.52	6.94	26.58	−0.244	Chl	CZ
*PlCSD1-3*	Peony_Unigene_151283	145	15,318.57	6.95	10.21	−0.028	Chl, Cyt.	CZ
*PlCSD2-1*	Peony_Unigene_483257	221	22,713.7	6.1	20.01	0.011	Chl	CZ
*PlCSD2-2*	Peony_Unigene_424839	189	19,737.87	6.9	28.47	−0.514	Chl, Cyt.	CZ
*PlCSD3*	Peony_Unigene_299453	163	16,766.82	6.74	18.39	−0.209	Chl, Cyt.	CZ
*PlFSD1*	Peony_Unigene_433554	236	26,301.77	6.71	37.04	−0.43	Chl	IMA, IMC
*PlFSD3*	Peony_Unigene_453151	261	29,972.54	8.17	41.09	−0.35	Chl	IMA, IMC
*PlMSD1*	Peony_Unigene_259976	237	26,472.27	8.98	48.54	−0.346	Mit	IMA, IMC
*PlMSD2*	Peony_Unigene_061444	248	27,771.29	6.04	26.19	−0.344	Mit	IMA, IMC

PI: isoelectric points; GRAVY: grand average of hydropathy; Chl: chloroplast; Cyt: cytoplasm; Mit: mitochondrion; CZ: Copper/zinc superoxide dismutase; IMA: Iron/manganese superoxide dismutases, alpha-hairpin domain; IMC: Iron/manganese superoxide dismutases, C-terminal domain.

**Table 3:** Secondary structure prediction of PlSOD proteins in *P. lactiflora*.

Gene	Alpha Helix (%)	Beta Turn (%)	Extended Strand (%)	Random Coil (%)
*PlCSD1-1*	10.31	5.15	30.41	54.12
*PlCSD1-2*	13.40	6.70	28.87	51.03
*PlCSD1-3*	8.11	7.43	34.46	50.00
*PlCSD2-1*	19.46	8.14	26.24	46.15
*PlCSD2-2*	12.17	6.35	32.28	49.21
*PlCSD3*	9.20	5.52	30.67	54.60
*PlFSD1*	32.63	3.81	16.53	47.03
*PlFSD3*	37.55	3.07	15.33	44.06
*PlMSD1*	52.74	4.22	12.66	30.38
*PlMSD2*	53.63	3.63	14.11	28.63

## Data Availability

The datasets used and/or analyzed are contained within the article and Supplementary Materials.

## Supplementary Materials

The following supporting information can be downloaded at: https://www.mdpi.com/article/10.3390/antiox13091128/s1, Table S1: RNA-seq data of *PlSOD* genes from leaves at six stages under natural high-temperature stress in herbaceous peony ‘Hang Baishao’; Table S2: details of the primers used in the qRT-PCR analysis of SOD genes of *P. lactiflora*; Table S3: SOD sequences in *P. lactiflora* and five other species; Table S4: SOD protein sequence similarities between *P. lactiflora* and *A. thaliana*. Sequence similarities (≥70) are shown in bold; Table S5: pairwise identity of SOD family genes in *P. lactiflora*.
